# Mediterranean Diet Reduces Risk of Incident Stroke in a Population With Varying Cardiovascular Disease Risk Profiles

**DOI:** 10.1161/STROKEAHA.117.020258

**Published:** 2018-09-20

**Authors:** Katherine E. Paterson, Phyo K. Myint, Amy Jennings, Lucy K.M. Bain, Marleen A.H. Lentjes, Kay-Tee Khaw, Ailsa A. Welch

**Affiliations:** 1 From the Department of Population Health and Primary Care (K.E.P., L.K.M.B., A.A.W.), Norwich Medical School, University of East Anglia, United Kingdom; 2 Department of Nutrition and Preventive Medicine (A.J.), Norwich Medical School, University of East Anglia, United Kingdom; 3 Department of Nutrition and Dietetics, Norfolk & Norwich University NHS Foundation Trust, United Kingdom (K.E.P.); 4 School of Medicine, Medical Sciences and Nutrition, University of Aberdeen, United Kingdom (P.K.M.); 5 Clinical Gerontology Unit, Department of Public Health and Primary Care, University of Cambridge, United Kingdom (M.A.H.L., K.-T.K.).

**Keywords:** cardiovascular disease, diet, epidemiology, stroke, women

## Abstract

**Background and Purpose—:**

Although some evidence has found that the Mediterranean diet (MD) is protective for stroke risk, few studies have investigated whether this relationship differs by sex or cardiovascular disease risk.

**Methods—:**

We investigated the relationship between adherence to the MD score, estimated using 7-day dietary diaries and risk of incident stroke in an observational prospective population-based cohort study of 23 232 men and women (54.5% women) aged 40 to 77 years who participated in the European Prospective Investigation into Cancer study in Norfolk, United Kingdom. Risk of incident stroke was calculated using multivariable Cox regression, in the whole population, and also stratified by sex and cardiovascular disease risk profile, using the Framingham risk score.

**Results—:**

During 17.0 years of follow-up (395 048 total person-years), 2009 incident strokes occurred. Risk of stroke was significantly reduced with greater adherence to the MD score (quartile 4 versus quartile 1 hazard ratio [HR], 0.83; 95% CI, 0.74–0.94; *P*-trend <0.01) in the whole population and in women (quartile 4 versus quartile 1 HR, 0.78; 95% CI, 0.65, 0.93; *P*-trend <0.01) but not in men (quartile 4 versus quartile 1 HR, 0.94; 95% CI, 0.79–1.12; *P*-trend =0.55). There was reduced risk of stroke in those at high risk of cardiovascular disease and across categories of the MD score (quartile 4 versus quartile 1 HR, 0.87; 95% CI, 0.76–0.99; *P*-trend =0.04). However, this was driven by the associations in women (quartile 4 versus quartile 1 HR, 0.80; 95% CI, 0.65–0.97; *P*-trend =0.02).

**Conclusions—:**

Greater adherence to the MD was associated with lower risk of stroke in a UK white population. For the first time in the literature, we also investigated the associations between the MD score in those at both low and high risk of cardiovascular disease. Although the findings in our study were driven by the associations in women, they have implications for the general public and clinicians for prevention of stroke.

## Abstract 2

Supplemental Digital Content is available in the text.

The burden of cerebrovascular disease is increasing with stroke being the third leading cause of disability-adjusted life-years, accounting for 6.24 million deaths annually worldwide.^1,2^ Around 90% of stroke risk is preventable and attributable to modifiable risk factors (RFs) including poor diet.^3^ Conformity to a Mediterranean diet (MD) has been found to be beneficial for stroke prevention, although the results of previous studies have varied because of differences in study design, existing cardiovascular disease (CVD) risk within populations, and different methods of dietary assessment.^4–6^ The beneficial effects of the MD are likely to be because of the overall effect or its constituent nutritional components on lowering blood pressure, lipids, and inflammation and improving metabolic health.^7^ The MD forms part of the dietary recommendations for guidelines for the primary prevention of stroke.^8,9^ However, only 2 prospective cohort studies have investigated the preventative effect of the MD on stroke in both men and women concurrently, despite recent guidelines suggesting that stroke prevention strategies should differ in men and women.^10^ This is because CVD risk profiles are sex specific, for example, women with diabetes mellitus experience a higher risk of stroke than men.^10,11^ Sex-specific research concerning the MD and risk of stroke has also been recommended because of the lack of studies with sufficiently robust outcome data.^11^

Although understanding the effect of the MD stroke risk is important for population prevention, previous studies have only investigated the effects of the MD either in populations with high risk of CVD or without stratification for CVD risk across whole populations.^5^ We are unaware of previous studies investigating the associations between the MD in those with low and high risk for CVD and the MD.

Key components of a traditional MD include olive oil as the main source of fat, high intakes of fish, fruit, vegetables, nuts, and legumes, and low meat and dairy consumption with moderate alcohol consumption.^12^ The CVD preventive effect of MD is conferred by the nutrient and bioactive compound contributions from the foods within this pattern. However, although the traditional MD is well established, the foods that contribute to it vary between Mediterranean and non-Mediterranean countries,^13^ resulting in different nutrient profiles for this dietary pattern. Moreover, food habits differ regionally even within Europe.^14^ This may impact on the median cut points of food and nutrient components that are used to form Mediterranean diet scores (MDSs) used in research and, therefore, the comparability between studies.

To our knowledge, although there have been studies that have examined the effect of an association between MD and incident stroke risk,^4^ only one previous study in the United Kingdom used food frequency questionnaire (FFQ) method to estimate food intake.^15^ Also, one small prospective cohort study (n=1849) previously used a 7-day diet diary (7DD) method^16^ despite this approach being considered more precise than FFQs for estimating food and nutrient intakes.^17^ FFQs are known to provide differing estimates of food components in comparison to food diaries, thus potentially distorting population median intake estimates of foods. For example, intakes of vegetables are ≈100 g/d higher when derived from FFQs compared with food diaries.^18^

The primary aims of our study were to examine whether greater adherence to MD was associated with reduced risk of incident stroke in a prospective cohort study of middle and older aged people. Given the previous research, our aims were first to understand whether the associations with the MD differed between men and women, second to understand these associations according to categories of CVD risk, and finally to understand whether the individual nutritional components of the MD were associated with risk of stroke.

## Methods

Data will be available on request from the corresponding author, subject to approval by the European Prospective Investigations into Cancer and Nutrition (EPIC) Norfolk steering committee.

The current prospective cohort study includes 23 232 voluntary participants from a population-based cohort of 25 639 men and women from EPIC-Norfolk, the UK arm of the multicenter European Prospective Investigation into Cancer study (Figure I in the online-only Data Supplement). The EPIC-Norfolk study design and the characteristics of the cohort have been described fully elsewhere.^19^ Briefly, all people aged between 40 and 79 years registered with 35 collaborating general practice surgeries in the Norfolk area were invited to the study by mail between 1993 and 1997. Participants who consented to the study completed a Health and Lifestyle Questionnaire and subsequently attended a health examination. The cohort is representative of a UK population, although current smoking levels were marginally lower than that reported in other UK surveys.^19^

### Baseline Measurements

At baseline, participants were asked to complete 7DDs for all foods and drinks consumed.^20^ Full details on 7DD methodology in addition to the collection of health and lifestyle information and anthropometric and biological measurements are available in the online-only Data Supplement. For the current analyses, we defined the MD using the modified MDS, frequently described as a traditional MD.^13^ In calculating this score, one point is given for intakes of each protective item in the score at or above the sex-specific median for the study population, and zero is given for amounts below the sex-specific median. Protective items are fruit and nuts, vegetables, legumes, cereals (including bread and potatoes), fish, and a higher unsaturated fat:saturated fat ratio. For dairy and meat and eggs, intakes at or above the sex-specific median intake received 0 point; for intakes below the sex-specific median, 1 point was given. Women received 1 point for intakes of alcohol between 5 and 25 g/d (4.4–21.9 U/wk) and men who drank between 10 and 50 g/d (8.8–43.7 U/wk) alcohol received 1 point; otherwise, men and women were given 0 points for other amounts of alcohol. Thus, a range of scores between 0 and 9 was possible.

### Outcome Ascertainment

Linkage with hospital records and death certificate information determined nonfatal and fatal stroke incidence. Stroke outcome data were available for almost 100% of the participants apart from a few individuals who died abroad (notified by relatives) and for whom we do not have a cause of death. These participants represent 1.4% of deaths within the cohort. *International Classification of Disease* codes ICD-9 430–448/ICD-10, I60-69 were classed as stroke. In the United Kingdom, all individuals have a unique National Health Service ID number, and the National Health Service is used by the whole population. This investigation includes incident strokes during follow-up (FU) until March 31, 2015. This outcome ascertainment method has been validated for stroke in EPIC showing high sensitivity and specificity.^21^

### Ethics

All participants gave written informed consent to the study. Norfolk District Health Authority Ethics Committee gave ethical approval.

### Statistical Analysis

Statistical analyses were performed using Stata version 11 (StataCorp. 2009. Stata Statistical Software: Release 14. StataCorp LP, College Station, TX).

We compared the baseline characteristics and unadjusted dietary intakes of men and women at baseline using 2 sample *t* tests with equal variance for continuous data or χ^2^ tests for categorical data. We also compared the baseline characteristics across sex-specific quartiles of MDS using ANOVA for continuous data or χ^2^ tests for categorical data.

Hazard ratios (HRs) and 95% CIs were determined for incident stroke risk in relation to quartiles of the MDS using Cox regression. For these analyses, we report the HRs, stratified by sex, from unadjusted and 3 adjusted models. Model 1: sex, age, body mass index, educational attainment, physical activity, smoking status, material deprivation using the Townsend Index, energy intake, and alcohol intake. Model 2: model 1+baseline serum total cholesterol, baseline myocardial infarction (MI) or diabetes mellitus, and family history of stroke or MI. Model 3: Model 2+systolic blood pressure, antihypertensive use, and aspirin use for >3 months. We also used Cox regression to examine the HRs for incident stroke comparing participants with intakes above and below the median for individual components of the MDS, with covariates as model 3 above.

To investigate the effect of MD adherence in individuals with different existing cardiovascular risk profiles, we performed further stratified analysis. We calculated the 10-year cardiovascular risk of participants using the traditional Framingham risk score^22^ and stratified participants into 2 groups: low risk (Framingham risk score below the median) and high risk (Framingham risk score above the median). HRs and 95% CIs were determined for incident stroke risk across quartiles of the MDS within these groups using Cox regression. Models were adjusted for sex, age, body mass index, educational attainment, physical activity, smoking status, material deprivation using Townsend deprivation index, energy intake, alcohol intake, baseline MI, and family history of stroke or MI. We also stratified these analyses by sex.

## Results

Of the 23 232 individuals aged 40 to 77 years at baseline (mean: 59.1±9.3 years), 54.5% were women. There were 2009 incident strokes (men n=961 and women n=1048) during FU (mean: 17.0±4.6 years; 395 048 total person-years). At baseline, systolic and diastolic blood pressure was significantly higher in men than in women by 3.6 mm Hg (SE: 0.2) and 3.5 mm Hg (SE: 0.1; *P*<0.001 for both), respectively. Mean MDS was 4.4 (SD: 1.6) in men and 4.2 (SD: 1.6) in women (*P*<0.01; Table 1). There was a significant trend for lower prevalence of current smoking and higher physical activity levels by increasing quartiles of MDS for both men and women. In women only, higher adherence to the MDS was also associated with lower age (*P*<0.01), body mass index (*P*<0.01), use of antihypertensive medication (*P*<0.01) and deprivation (*P*=0.03), and higher education levels (*P*<0.01; Table II in the online-only Data Supplement).

**Table 1.**
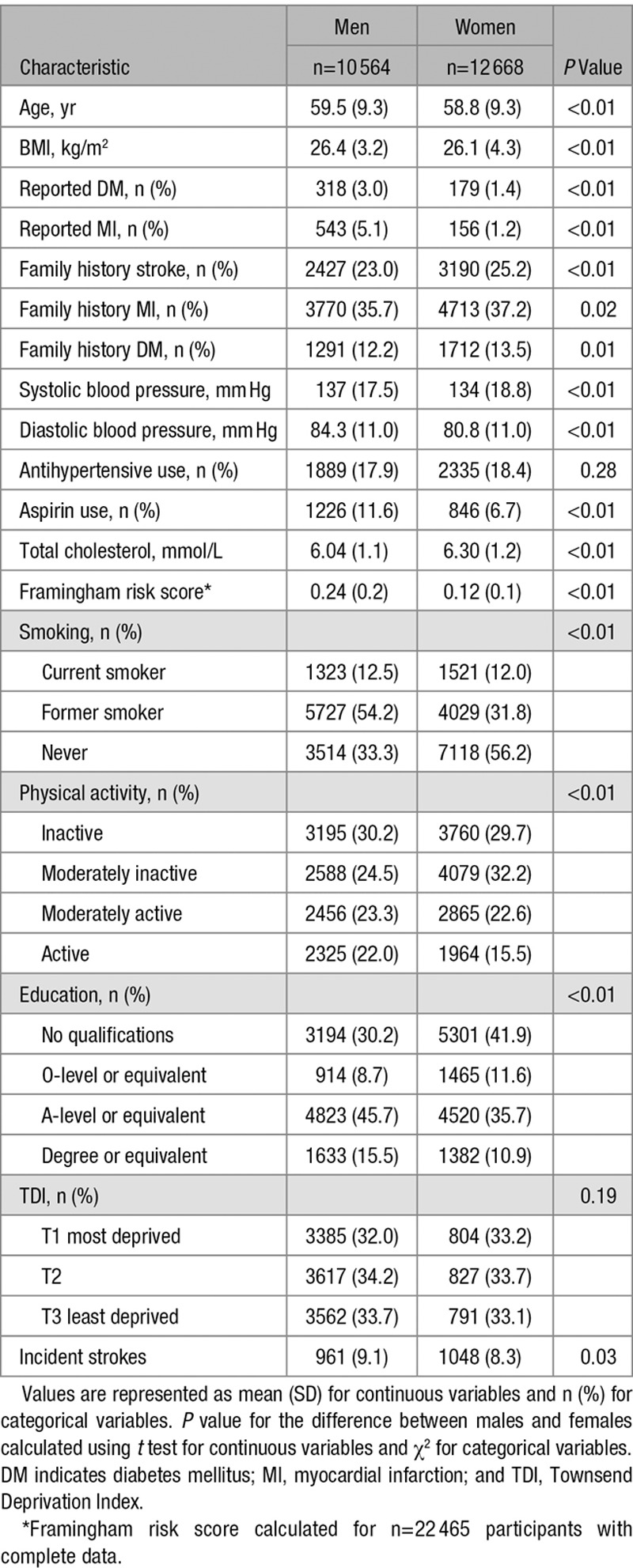
Baseline Characteristics in 23 232 Men and Women Aged 40 to 77 Years in the EPIC-Norfolk Cohort

In multivariable analysis (Table 2), in the whole study population, there was a significant inverse trend for reduced stroke risk across increasing quartiles of the MDS (quartile 4 versus quartile 1 HR, 0.83; 95% CI, 0.74–0.94; *P*-trend <0.01). After stratification by sex, these associations were only apparent in women (quartile 4 versus quartile 1 HR, 0.78; 95% CI, 0.65–0.93; *P*-trend <0.01). Additionally, moderate MDS (quartile 3 versus quartile 1) was associated with a 20% significant stroke risk reduction in women (HR, 0.80; 95% CI, 0.67–0.95; *P*<0.05) but not in men. Sensitivity analysis with additional adjustment for hormone replacement therapy and menopausal status in women did not markedly change the results (data not shown).

**Table 2.**
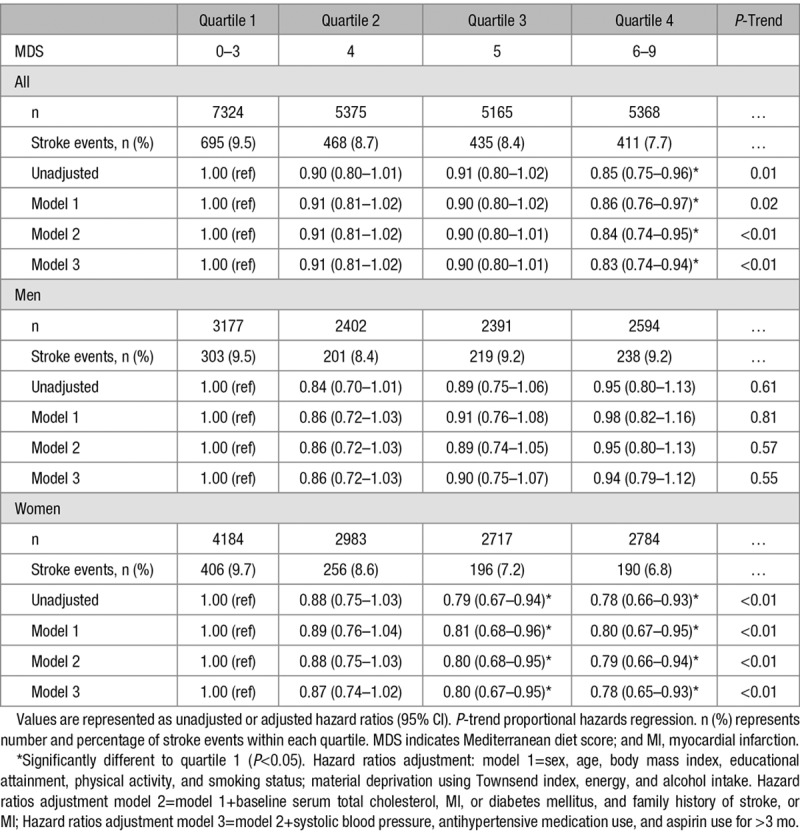
Associations Between Baseline MDS and Incident Stroke Risk in 23 232 Men and Women, Aged 40 to 77 Years in the EPIC-Norfolk Cohort

As shown in Table 3, we found higher adherence to the MDS to be associated with reduced stroke risk in those participants at high cardiovascular risk (Framingham risk score above the cohort-specific median). Participants with highest adherence to the MDS had a 13% reduced risk of stroke compared with those in the lowest MDS quartile (quartile 4 versus quartile 1 HR, 0.87; 95% CI, 0.76–0.99; *P*-trend =0.04). No significant associations were observed in the subgroup with low cardiovascular risk (quartile 4 versus quartile 1 HR, 0.77; 95% CI, 0.58–1.02; *P*-trend =0.09). After stratification by sex, it was apparent that the associations found in the high-risk group were driven by the findings in women. There was a reduction of 20% in the women (quartile 4 versus quartile 1 HR, 0.80; 95% CI, 0.65–0.97; *P*-trend =0.02). However, the associations in the low-risk groups were not significant in either sex.

**Table 3.**
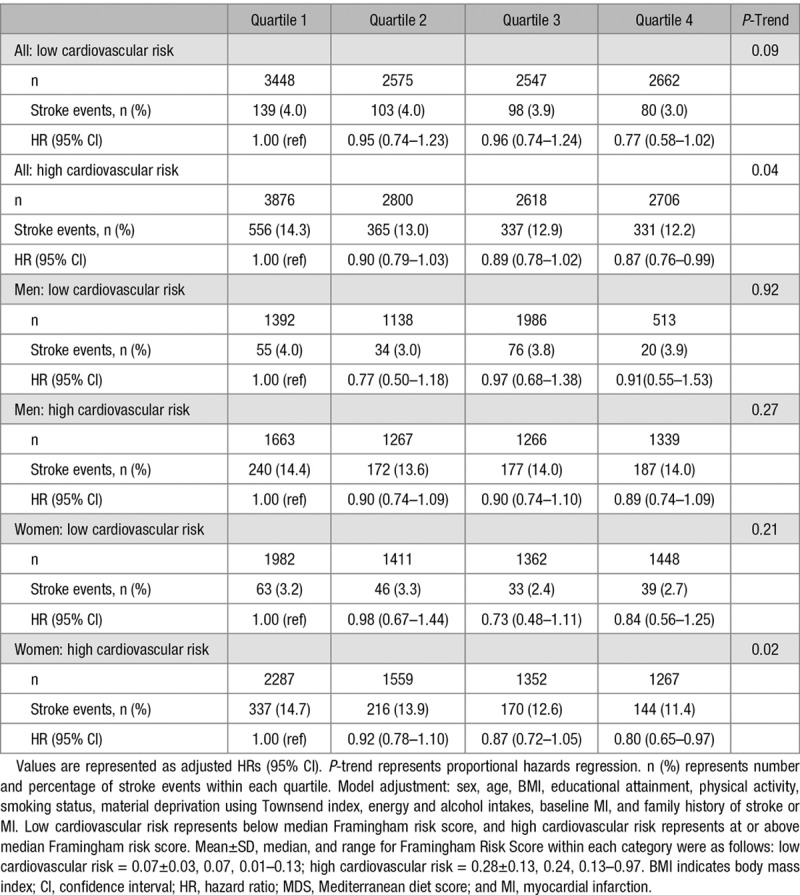
Associations Between Baseline MDS and Incident Stroke Risk in 22 465 Men and Women, Aged 40 to 77 Years in the EPIC-Norfolk Cohort Stratified by Framingham Risk Score

Analysis of the individual components of the MDS highlighted that the reduction in stroke risk seemed to be driven by the vegetable (HR, 0.87; 95% CI, 0.80–0.96) and alcohol (HR, 0.90; 95% CI, 0.82–0.99) components of the score (Table III in the online-only Data Supplement).

## Discussion

Our study found that greater adherence to the MD was associated with lower risk of stroke in a UK white population. We found these significant beneficial associations in the whole population with an HR 0.83 (95% CI, 0.74–0.94) between higher and lower adherence to the MD (quartile 4 versus quartile 1). These associations were also significant in women (HR, 0.78; 95% CI, 0.65–0.93), and although the HR was lower in men (HR, 0.94; 95% CI, 0.79–1.12), the associations were not significant. We also found that risk of stroke differed by risk of CVD, classified by the Framingham risk score, as well as by the MD in the whole population (quartile 4 versus quartile 1 HR, 0.87; 95% CI, 0.76–0.99; *P*-trend =0.04) and in women (quartile 4 versus quartile 1 HR, 0.80; 95% CI, 0.65–0.97; *P*-trend =0.02). To our knowledge, our study is the first to investigate and find differences in risk of stroke, not only according to the MD but also in those with low and high risk for CVD, and in men and women. Although our findings of a protective effect of the MD on risk of stroke were driven by women, they have implications for the general public and clinicians for the prevention of stroke.

We observed few significant associations with individual MD components, indicating that the effects of the MD may be attributable to the synergistic or additive effects of the individual foods or nutrients within the MD^12^ and that the overall MD pattern was most important in relation to stroke risk.

Although previous systematic reviews and meta-analyses of RCTs and observational studies have shown significant inverse associations in relation to MD adherence and risk of incident stroke, with studies finding a relative risk of 0.66 (95% CI, 0.48–0.92) and 0.73 (95% CI, 0.59–0.91), only 2 observational studies investigated men and women separately in the same cohort.^5,6,23,24^ It is important to understand sex differences in exposure to the MD because women have unique stroke RFs,^11^ which include postmenopausal hormone use, hormone status changes, pregnancy, preeclampsia, gestational diabetes mellitus, and oral contraceptive use. The prevalence of other RFs differs between men and women with hypertension, diabetes mellitus, atrial fibrillation, and migraine with aura^11^ being more prevalent in women.

Of the 2 other studies investigating sex differences in associations of stroke with the MD, one observational study in Hong Kong Chinese showed a significant inverse association with higher adherence to MD in men but not in women,^23^ whereas the other, in Greece, found reductions in incident stroke^24^ in women, only. It is not clear why we found differences in the associations between the MD and risk of stroke in men and women. However, we postulate that the components of the MD may influence the risk of stroke in women more than in men. We are also aware that the different subtypes of stroke may differ in men between sexes and may lead to heterogeneity of response to the MDS.^24^ We were unable to test this hypothesis in our cohort because of low incidence. However, this deserves further study in the future.

One previous study in the United Kingdom found a nonsignificant inverse association (HR, 0.96; 95% CI = 0.90–1.02) with greater MD adherence and incident stroke.^15^ However, this study did not stratify by sex, the MD was based on FFQs, and average FU was 12.2 years. We build on their analyses by including sex stratified analyses, using 7DDs with a longer FU period.

### Study Strengths

The strengths of our study include the prospective design, the use of a comprehensive 7DD, robust outcome assessment method, with few individuals lost to FU, and, besides smoking status, the population is representative of the United Kingdom.^19^ Another of our study’s advantages include longer FU of 17 years than all except one study in women, only. In addition, using 7DD to measure dietary/nutritional intake is more precise than FFQs, which may overestimate the important components of MD.^17^ We controlled for and measured potential nondietary confounding factors that affect stroke risk, namely, directly measured BP and cholesterol at baseline. Also, we had sufficient power to detect an effect of adherence to the MD on incident stroke in the population and in women when measured separately.

### Limitations

Dietary assessment was based on one assessment at baseline, which does not exclude the possibility that dietary changes or other treatments may occur subsequently, which would alter incident stroke risk. In addition, we did not control for some stroke RFs, for example, atrial fibrillation. Nevertheless, we controlled for major stroke RFs such as previous MI, BP, and vascular diseases. The MDS uses relative rather than absolute values for MD components. This limits comparison with other studies using the same score, as median intakes may be distinct in different populations. Our population was predominantly white, and, therefore, our results may not apply to other ethnic groups.

### Summary

Greater adherence to the MD was associated with lower risk of stroke in a UK white population, which was attributable to the overall MD pattern, not its individual components. For the first time in the literature, we also investigated the potential benefits of the MD in people at low and high risk of CVD, in men and in women. Although the findings in our study were driven by the associations in women, they have implications for the general public and clinicians for prevention of stroke.

## Acknowledgments

We thank participants, general practitioners, and EPIC-Norfolk staff.

## Sources of Funding

EPIC-Norfolk is supported by the Medical Research Council program grants (G0401527 and G1000143) and Cancer Research UK program grants (C864/A8257 and C864/A14136).

## Disclosures

None.

## Supplementary Material
