# Supplementary material for: Mediterranean Diet Reduces Risk of Incident Stroke in a Population With Varying Cardiovascular Disease Risk Profiles
Source: Stroke. 2018 Sep 20;49(10):2415–20. doi: 10.1161/STROKEAHA.117.020258 (PMC6159687; doi:10.1161/STROKEAHA.117.020258)
Supplement: Supplementary file 1 [file str-49-2415-s001.pdf]

Supplemental Material

Title: The Mediterranean Diet Reduces Risk of Incident Stroke in a Population with Varying Cardiovascular Disease Risk Profiles

Katherine E. Paterson, MRes; Phyo K.Myint, MD; Amy Jennings, PhD; Lucy KM Bain, PhD; Marleen J. Lentjes, PhD; Kay-Tee Khaw, MBBChir, MD; Ailsa A. Welch, PhD
Corresponding author: A.A Welch Email:a.welch@uea.ac.uk. Phone: +441603591950

Contents

Supplemental Methods
Supplemental Tables: 3
Supplemental Figure: 1
Supplemental References

Supplemental methods

Assessment of conformity to Mediterranean Dietary pattern (MD) using 7-day diet diaries

Food intake was assessed using pre-structured 7-day diet diaries[1,2]. Portion sizes were estimated by participants who made use of household measures, manufacturers' information or a series of colour photographs provided in the 7-day diary booklet. Using specifically created DINER (Data Into Nutrients for Epidemiological Research) software, data from diaries were coded and entered by trained nutritionists using a standard validated procedure[2]. The weights of consumed foods with similar characteristics were grouped together to obtain food groups and these food groups were subsequently summed to represent the components of the MDS (see Tables I & III). The weights of the food groups represent 'aggregated' food weights for mixed dishes i.e. they were not evaluated at the ingredient level, but at the level of consumption. So for example, the weight of the meat dishes included the weight of non-meat food items such as vegetables or other ingredients. Therefore, the food weights for meats, fish and offal were likely to be overestimated and the weights for fruits and vegetables more likely to be underestimated [1,2].

The modified Mediterranean diet score (MDS) was developed for use in populations where intake of monounsaturated fatty acids (MUFA) from olive oil is minimal and vegetable oils such as sunflower oils, rich in n-6 polyunsaturates, are the main sources of fat.

Health and Lifestyle information

Participants were asked to complete a Health and Lifestyle Questionnaire which included questions on physical activity, smoking status, educational level, occupation, pre-existing medical conditions (including stroke, diabetes and Myocardial Infarction) and medication use. The physical activity questionnaire asked about work and leisure time physical activity in the last year. Participants were classified as either active, moderately active, moderately inactive or inactive [3]. For smoking status participants were classified as non-, current- or former-smokers. Missing smoking status information was reclassified as 'current smoking' (n=176) to attenuate under reporting bias. Educational status was based on the highest qualification attained and was categorized into 4 groups as follows: degree or equivalent, A-level or equivalent, O-level or equivalent, and no qualifications. O-level indicates educational attainment to the equivalent of completion of schooling to the age of 15 y, and A-level indicates educational attainment to the equivalent of the completion of schooling to the age of 17 y. Townsend deprivation scores were divided into tertiles with cut off points of 7.0, −1.8, and −3.2 respectively with larger numbers indicating higher level of deprivation. Participants with missing data on aspirin use in the previous three months (n=3548) were recoded as non-users.

Anthropometric and biological measurements

Uniform procedures using trained personnel at participants' GP surgeries or the study clinic were used for determining weight, height, blood pressure (BP) and serum cholesterol levels [3]. A free standing stadiometer was employed to determine height to the nearest mm. Weight

was measured without shoes and with light clothing and recorded with 0.2 kg precision. Body Mass Index was calculated as weight in kg / (height in meters squared).

Two BP readings were taken after participants had been sitting for 3 minutes. An Accutorr Sphygmomanometer (Datascope, UK) was used and the participants' held their arm horizontally at the point of the mid sternum[3]. Serum cholesterol was measured from non-fasting venous blood sample.

Supplemental results

We observed a significant inverse relationship between consumption of vegetables and incident stroke risk in the whole cohort (HR 0.87; 95% CI 0.80, 0.96; *P*-trend = 0.01) and in men only (HR 0.87; 95% CI 0.76, 0.99; *P*-trend = 0.03). Moderate alcohol consumption was also associated with significantly reduced stroke risk in the whole cohort (HR 0.90; 95% CI 0.82, 0.99; *P*-trend = 0.03) which was driven by the associations for women (HR 0.83; 95% CI 0.72, 0.95; *P*-trend = 0.01). No other significant associations were observed. **(Table III)**

Table I. Baseline dietary intakes of 23,232 men and women aged 40-77 years stratified by quartiles of the Mediterranean Diet Score in the EPIC-Norfolk cohort

		Quartile 1	Quartile 2	Quartile 3	Quartile 4	*P*
	Median (IQR)	Mean (SD)	Mean (SD)	Mean (SD)	Mean (SD)	
**Men**						
n=10,564		3177	2402	2391	2594	-
MDS components						
Mediterranean diet score	4 (3-6)	2.5	4	5	6.5	
Fruit and nuts (g/d)	120 (49.1-209)	95.8 (112)	137 (125)	165 (136)	201 (139)	<0.01
Vegetables (g/d)	96.0 (59.9-143)	84.3 (67.3)	101 (68.7)	116 (73.7)	139 (74.9)	<0.01
Legumes (g/d)	0 (0-27)	10.5 (24.9)	16.6 (32.9)	18.3 (26.8)	24.9 (30.5)	<0.01
Meat and eggs (g/d)	137 (93.9-186)	170 (91.0)	148 (79.6)	136 (71.8)	123 (68.1)	<0.01
Dairy (g/d)	258 (164-376)	331 (185)	296 (178)	267 (162)	243 (144)	<0.01
Cereal and potatoes (g/d)	404 (328-491)	373 (122)	409 (134)	433 (137)	464 (130)	<0.01
Fish (g/d)	32.9 (12.6-59.3)	24.4 (31.6)	38.3 (37.7)	47.0 (42.2)	60.1 (42.1)	<0.01
Alcohol (g/d)	9.3 (0.8-24.7)	15.6 (25.6)	16.3 (21.9)	17.0 (20.1)	18.8 (16.9)	<0.01
Fat ratio (UFA: SFA)	1.46 (1.25-1.69)	1.32 (0.30)	1.45 (0.34)	1.56 (0.35)	1.67 (0.35)	<0.01
Nutrients						
Energy intake (kcal/d)	2227 (1896-2571)	2177 (547)	2241 (527)	2264 (522)	2328 (492)	<0.01
Fat (g/d)	84.2 (67.9-102)	86.0 (27.0)	87.1 (27.5)	86.3 (26.6)	86.3 (25.9)	0.48
Carbohydrate (g/d)	270 (224-317)	261 (75.4)	272 (72.1)	278 (73.1)	289 (69.6)	<0.01
Protein (g/d)	81.1 (70.2-93.0)	80.3 (19.2)	82.1 (18.8)	82.6 (17.9)	84.9 (17.6)	<0.01
Fibre (g/d)	15.3 (12.0-19.4)	13.6 (5.1)	15.7 (5.6)	16.9 (5.9)	19.2 (6.0)	<0.01
Magnesium (mg/d)	314 (261-373)	297 (84.7)	318 (90.2)	330 (89.2)	354 (89.5)	<0.01
Potassium (mg/d)	3414 (2933-3972)	3222 (777)	3419 (783)	3523 (803)	3756 (765)	<0.01
Calcium (mg/d)	894 (718-1090)	930 (312)	928 (311)	910 (294)	920 (266)	0.07
Vitamin C (mg/d)	73.4 (49.6-110)	70.8 (49.2)	83.0 (49.9)	89.8 (51.1)	103 (53.2)	<0.01
Sodium (mg/d)	3125 (2598-3695)	2641 (836)	2738 (864)	2801 (852)	2913 (850)	<0.01

**Women**						
n=12,668		4184	2983	2717	2784	-
MDS components						
Mediterranean diet score	4 (3-5)	2.5	4	5	6.5	
Fruit and nuts (g/d)	153 (81.4-244)	129 (114)	171 (127)	194 (131)	235 (142)	<0.01
Vegetables (g/d)	98.3 (61.9-144)	86.1 (63.5)	106 (68.6)	121 (69.2)	142 (72.0)	<0.01
Legumes (g/d)	0 (0-19.5)	6.4 (16.8)	9.9 (19.3)	13.1 (21.8)	17.8 (21.7)	<0.01
Meat and eggs (g/d)	104 (68.3-144)	129 (70.4)	111 (62.2)	102 (58.3)	89.8 (51.2)	<0.01
Dairy (g/d)	242 (156-348)	305 (166)	265 (154)	249 (148)	224 (132)	<0.01
Cereal and potatoes (g/d)	302 (241-370)	283 (99.8)	306 (104)	325 (106)	348 (103)	<0.01
Fish (g/d)	30.0 (12.9-51.4)	22.5 (25.7)	34.4 (32.6)	41.1 (32.5)	50.7 (32.1)	<0.01
Alcohol (g/d)	3.0 (0-11.4)	7.3 (13.7)	8.0 (12.5)	8.6 (11.7)	8.9 (9.74)	<0.01
Fat ratio (UFA: SFA)	1.44 (1.24-1.66)	1.31 (0.31)	1.46 (0.35)	1.54 (0.35)	1.67 (0.34)	<0.01
Nutrients						
Energy intake (kcal/d)	1688 (1429-1947)	1654 (402)	1677 (384)	1733 (398)	1755 (372)	<0.01
Fat (g/d)	63.5 (50.3-77.6)	65.2 (21.2)	64.1 (20.3)	65.7 (21.2)	64.1 (20.2)	<0.01
Carbohydrate (g/d)	211 (176-248)	203 (53.6)	210 (52.4)	219 (54.4)	227 (52.0)	<0.01
Protein (g/d)	64.8 (56.0-74.3)	64.2 (14.6)	65.0 (14.3)	66.3 (14.4)	67.0 (12.8)	<0.01
Fibre (g/d)	13.6 (10.8-16.8)	12.1 (4.4)	13.8 (4.6)	15.0 (4.8)	16.8 (5.0)	<0.01
Magnesium (mg/d)	260 (218-308)	248 (69.1)	262 (69.2)	276 (74.4)	295 (74.0)	<0.01
Potassium (mg/d)	2958 (2539-3421)	2805 (668)	2949 (669)	3092 (682)	3272 (671)	<0.01
Calcium (mg/d)	746 (598-911)	782 (259)	762 (251)	767 (248)	757 (227)	<0.01
Vitamin C (mg/d)	79.8 (53.8-116)	76.0 (46.9)	87.9 (48.4)	95.7 (48.5)	107 (51.2)	<0.01
Sodium (mg/d)	2365 (1969-3786)	2329 (662)	2385 (655)	2441 (651)	2525 (646)	<0.01

Values are median or mean (SD). UFA: SFA, Unsaturated: saturated fatty acids
Values are mean (SD) for UFA: SFA, Unsaturated: saturated fatty acids

Table II. Baseline characteristics* in 23,232 men and women aged 40-77 year stratified by quartiles of the Mediterranean Diet Score in the EPIC-Norfolk cohort

	Quartile 1	Quartile 2	Quartile 3	Quartile 4	*P*
**MDS range**	0-3	4	5	6-9	
**Men**					
n=10564	3177	2402	2391	2594	-
Age (years)	59.6 (9.5)	59.6 (9.4)	59.7 (9.2)	59.1 (9.0)	0.07
BMI (kg/m^2)	26.5 (3.3)	26.5 (3.2)	26.6 (3.3)	26.4 (3.2)	0.16
Reported DM	72 (2.3 %)	69 (2.9%)	89 (3.7%)	88 (3.4%)	0.01
Reported MI	159 (5.0 %)	115 (4.8%)	110 (4.6%)	159 (6.1%)	0.06
Family history stroke	701 (22.1 %)	545 (22.7%)	568 (23.8%)	613 (23.6%)	0.38
Family history MI	1129 (35.5%)	869 (36.2%)	853 (35.7%)	919 (35.4%)	0.95
Family history DM	358 (11.3%)	295 (12.3%)	296 (12.4%)	342 (13.2%)	0.17
Systolic blood pressure (mm Hg)	137.2 (17.5)	137.5 (17.9)	137.3 (17.3)	137 (17.3)	0.78
Diastolic blood pressure (mm Hg)	84.4 (10.8)	84.5 (11.5)	84.2 (10.8)	84.1 (11.0)	0.44
Antihypertensive use	554 (17.4%)	406 (16.9%)	432 (18.1%)	497 (19.2%)	0.18
Aspirin use	342 (10.8%)	273 (11.4%)	276 (11.6%)	335 (12.9%)	0.08
Total cholesterol (mmol/L)	6.0 (1.1)	6.0 (1.1)	6.1 (1.1)	6.0 (1.1)	0.24
Smoking: current smoker	556 (17.5%)	289 (12.0%)	247 (10.3%)	231 (8.9%)	<0.01
former smoker	1640 (51.6%)	1300 (54.1 %)	1332 (55.7%)	1455 (56.1%)	
never	981 (30.9%)	813 (33.9%)	812 (34.0%)	908 (35.0%)	
Physical activity: inactive	1077 (33.9%)	724 (30.1%)	719 (30.1%)	675 (26.0%)	<0.01
moderately inactive	760 (23.9%)	587 (24.4%)	599 (25.0%)	642 (24.7%)	
moderately active	717 (22.6%)	571 (23.8%)	554 (23.2%)	663 (25.6%)	
active	623 (19.6%)	520 (21.6%)	519 (21.7%)	663 (25.6%)	
Education: no qualifications	1017 (32%)	690 (28.7%)	736 (30.8%)	751 (28.9%)	0.05
O-Level or equivalent	289 (9.1%)	212 (8.8%)	210 (8.8%)	203 (7.8%)	
A-Level or equivalent	1407 (44.3%)	1112 (46.3%)	1094 (45.7%)	1210 (46.7%)	
degree or equivalent	464 (14.6%)	388 (16.15%)	351 (14.7%)	430 (16.6%)	
TDI: T1 most deprived	1043 (32.8%)	773 (32.2%	751 (31.4%)	818 (31.5%)	0.57

T2	1069 (33.6%)	844 (35.1%)	835 (34.9%)	869 (33.5%)	
T3 least deprived	1065 (33.5%)	785 (32.7%)	805 (36.7%)	907 (35.0%)	
MDS	2.5 (0.72)	4 (0)	5 (0)	6.5 (0.70)	-
**Women**					
n=12668	4184	2983	2717	2784	
Age (years)	59.4 (9.5)	59.1 (9.3)	58.4 (9.2)	58.1 (8.9)	<0.01
BMI (kg/m^2)	26.3 (4.4)	26.3 (4.2)	26.0 (4.0)	25.8 (4.2)	< 0.01
Reported DM	57 (1.4%)	38 (1.3%)	28 (1.0%)	56 (2.0%)	0.02
Reported MI	52 (1.2 %)	35 (1.2%)	33 (1.2%)	36 (1.3%)	0.98
Family history stroke	1020 (24.4%)	771 (25.8%)	722 (26.6%)	677 (24.3%)	0.11
Family history MI	1524 (36.4%)	1067 (35.8%)	1023 (37.6%)	1099 (39.5%)	0.02
Family history DM	534 (12.8%)	396 (13.3%)	354 (13%)	428 (15.4%)	0.01
Systolic blood pressure (mm Hg)	134.0 (19.0)	133.8 (18.9)	133.4 (18.4)	133.2 (18.6%)	0.24
Diastolic blood pressure (mm Hg)	81.0 (11.2)	80.7 (11.0)	80.7 (10.8)	80.8 (11.1)	0.65
Antihypertensive use	806 (19.3%)	573 (19.2%)	457 (16.8%)	499 (17.9%)	0.04
Aspirin use	295 (7.0 %)	204 (6.8%)	162 (6.0%)	185 (6.7%)	0.35
Total cholesterol (mmol/L)	6.4 (1.2)	6.3 (1.2)	6.3 (1.2)	6.2 (1.2)	<0.01
Smoking: current smoker	661 (15.8%)	366 (12.3%)	282 (10.4%)	212 (7.6%)	<0.01
former smoker	1282 (30.6%)	922 (30.9%)	878 (37.3%)	947 (34.0%)	
never	2241 (53.6%)	1695 (56.8%)	1557 (57.3%)	1625 (58.4%)	
Physical activity: inactive	1452 (34.7%)	905 (30.3%)	726 (26.7%)	677 (24.3%)	<0.01
moderately inactive	1297 (31%)	981 (32.9%)	877 (32.3%)	924 (33.2%)	
moderately active	891 (21.3%)	636 (21.3%)	643 (23.7%)	695 (25.0%)	
active	544 (13.0%)	461 (15.5%)	471 (17.3%)	488 (17.5%)	
Education: no qualifications	1820 (43.5%)	1287 (43%)	1142 (42.0%)	1052 (37.8%)	<0.01
O-Level or equivalent	452 (10.8%)	355 (11.9%)	320 (11.8%)	338 (12.1%)	
A-Level or equivalent	1479 (35.4%)	1056 (35.4%)	932 (34.3%)	1053 (37.8%)	
degree or equivalent	433 (10.3%)	285 (9.5%)	323 (11.9%)	341 (12.3%)	
TDI: T1 most deprived	1443 (34.5%)	994 (33.3%)	904 (33.3%)	860 (30.9%)	0.03
T2	1359 (32.5%)	1040 (34.9%)	912 (33.6%)	959 (34.5%)	
T3 least deprived	1382 (33.0%)	949 (31.8%)	901 (33.2%)	965 (34.7%)	

MDS	2.5 (0.73)	4 (0)	5 (0)	6.5 (0.68)	-

Values are mean (SD) for continuous variables and n= (%) for categorical variables. *P*-value for the difference between continuous data by quartile of the MDS using analysis of variance and chi-square for categorical variables.
DM indicates diabetes mellitus; MDS, Mediterranean diet score; MI, myocardial infarction; TDI, Material deprivation using Townsend Index;

Table III. Associations between individual components of the Mediterranean Diet Score and incident stroke risk in 23,232 men and women, aged 40-77 years in the EPIC-Norfolk cohort

	All (n=23,232)		Men (n=10,564)		Women (12,668)	
	**Hazard ratio (95% CI)**	***P*=**	**Hazard ratio (95% CI)**	***P*=**	**Hazard ratio (95% CI)**	***P*=**
Fruit and nuts	0.93 (0.85, 1.02)	0.10	0.99 (0.87, 1.13)	0.87	0.91 (0.81, 1.03)	0.15
Vegetables	0.87 (0.80, 0.96)	0.03	0.87 (0.76, 0.99)	0.03	0.90 (0.79, 1.02)	0.09
Legumes	0.94 (0.86, 1.02)	0.15	0.95 (0.84, 1.08)	0.47	0.92 (0.81, 1.04)	0.18
Meat and eggs	0.93 (0.85, 1.02)	0.14	0.94 (0.82, 1.07)	0.32	0.94 (0.83, 1.06)	0.31
Dairy	1.03 (0.94, 1.13)	0.51	1.02 (0.89, 1.17)	0.72	1.01 (0.88, 1.14)	0.94
Cereal and potatoes	0.99 (0.89, 1.11)	0.91	1.01 (0.88, 1.17)	0.87	0.98 (0.85, 1.12)	0.76
Fish	0.94 (0.86, 1.03)	0.20	0.97 (0.85, 1.10)	0.60	0.93 (0.82, 1.05)	0.21
Alcohol	0.90 (0.82, 0.99)	0.04	0.97 (0.85, 1.11)	0.64	0.83 (0.72, 0.95)	0.01
Fat ratio	1.03 (0.94, 1.13)	0.48	1.01 (0.97, 1.25)	0.14	0.95 (0.84, 1.08)	0.42

Values are the adjusted hazard ratios (95% CI) comparing participants with intakes above vs. below the median for each component except the Meat and eggs and Dairy components, which compare participants with intakes below vs. above the median. Each component was included in a separate model. *P*= difference between the two groups calculated using Cox Proportional Hazards Regression. Hazard ratios were adjusted for sex, age, body mass index, physical activity, smoking status, educational attainment, material deprivation, energy intake, alcohol intake, serum total cholesterol, baseline myocardial infarction or diabetes and family history stroke or myocardial infarction, systolic blood pressure, aspirin use and antihypertensive use. Alcohol intake was not included as a covariate in the alcohol model.

Figure I: Flow chart detailing participant recruitment for the European Prospective Investigation of Cancer Participants recruited at baseline with reasons for exclusion in the analysis

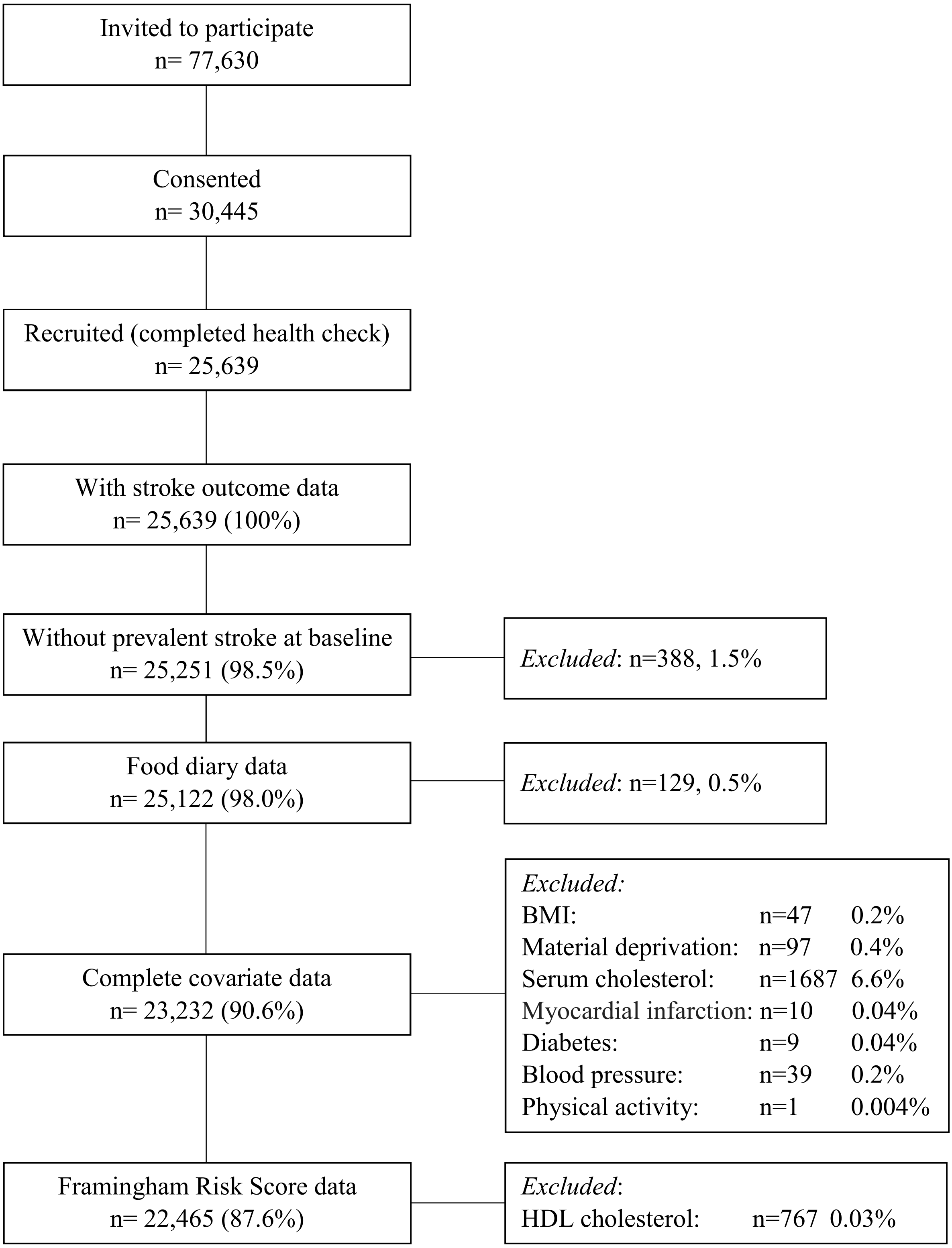

References

1. Lentjes MA, McTaggart A, Mulligan AA, Powell NA, Parry-Smith D, Luben RN, et al. Dietary intake measurement using 7 d diet diaries in british men and women in the european prospective investigation into cancer-norfolk study: A focus on methodological issues. *Br J Nutr*. 2014;111:516-526
2. Welch AA, McTaggart A, Mulligan AA, Luben R, Walker N, Khaw KT, et al. Diner (data into nutrients for epidemiological research) - a new data-entry program for nutritional analysis in the epic-norfolk cohort and the 7-day diary method. *Public Health Nutr*. 2001;4:1253-1265
3. Day N, Oakes S, Luben R, Khaw KT, Bingham S, Welch A, et al. Epic-norfolk: Study design and characteristics of the cohort. European prospective investigation of cancer. *Br J Cancer*. 1999;80 Suppl 1:95-103
